# Dynamic Evolution of Aroma Characteristics in Ripened Pu-Erh Tea During Industrial Fermentation: Insights from GC-MS and Flavor Wheel Analysis

**DOI:** 10.3390/foods15061014

**Published:** 2026-03-13

**Authors:** Yiqing Guan, Qiuyue Chen, Nianguo Bo, Dihan Yang, Fan Yang, Hongyan Gao, Xiaying Tao, Ping Liang, Guanghong Pan, Bei Cai, Yingling Zhou, Hao Zhang, Shaohua Peng, Lei Shi, Teng Wang

**Affiliations:** 1 The Key Laboratory of Agricultural Microbiome of Yunnan Province, Kunming 650201, China; dxcxygyq@126.com (Y.G.); 13669799142@163.com (Q.C.); 18487115352@163.com (N.B.); 15680783708@163.com (D.Y.); 18887400747@163.com (F.Y.); 14787855147@163.com (H.G.); xiayingtao666666@163.com (X.T.); 13337259484@163.com (P.L.); 15752328598@163.com (G.P.); 18708605279@163.com (B.C.); 15828552660@163.com (Y.Z.); 2 The National & Local Joint Engineering Research Center on Germplasm Innovation & Utilization of Chinese Medicinal Materials in Southwestern China, Kunming 650201, China; 3 College of Tea Science, Yunnan Agricultural University, Kunming 650201, China; 4 Xiaguan Tuocha (Group) Co., Ltd., Dali 671000, China; 13529922926@163.com (H.Z.); m13577855911@163.com (S.P.); 18314574872@163.com (L.S.); 5 College of Agronomy and Biotechnology, Yunnan Agricultural University, Kunming 650201, China

**Keywords:** ripened Pu-erh tea, industrial pile fermentation, volatile organic compounds, stage-dependent evolution, flavor wheel

## Abstract

Ripened Pu-erh tea (RPT) experiences notable aroma transformations during industrial pile fermentation, yet the stage-dependent evolution of key aroma compounds remains poorly understood. This study analyzed two independent industrial batches of RPT across three fermentation stages: raw material (RM), intermediate fermentation (IF), and final fermentation (FF). Using HS-SPME/GC-MS coupled with multivariate statistical analysis and relative odor activity values (rOAVs), 134 volatile organic compounds (VOCs) were identified, with hydrocarbons, alcohols, and esters as predominant classes. In total, 13 key aroma-active compounds (rOAVs > 1) were found to be major contributors to RPT’s characteristic aroma. During early fermentation, relative levels of VOCs responsible for fresh and green aromas (e.g., linalool, *D*-limonene) diminished, while those for woody and minty aromas (e.g., isophorone, methyl salicylate) increased. A flavor wheel was developed to illustrate the dynamic shifts in aroma profiles. This stage-resolved analysis offers new mechanistic insights into aroma formation, aiding in the optimization of aroma quality control and process standardization for RPT production.

## 1. Introduction

Tea remains the second most widely consumed beverage globally, surpassed only by water. Among the six major tea categories produced in China, dark tea is unique for its microbial-driven post-fermentation process, which systematically reshapes flavor chemistry and facilitates the formation of diverse bioactive metabolites [1,2]. Ripened Pu-erh tea (RPT), a flagship geographic indication product of dark tea, is produced exclusively in Yunnan Province from the fresh leaves of *Camellia sinensis* var. *assamica* [3,4]. Driven by its distinct sensory appeal and perceived health benefits, RPT has garnered significant market relevance across Asia, Europe, and North America [4,5,6]. The scale of this industry is underscored by the 2024 Development Report of the Yunnan Province Tea Industry, which recorded an annual production of 185,000 metric tons. Beyond its commercial value, RPT is recognized as a functional food, with substantial evidence from animal models and human trials validating its hypolipidemic, hypoglycemic, and gut microbiota-modulating effects [7].

The commercial success of RPT relies heavily on its distinctive sensory attributes, characterized by a brownish-red infusion, a “stale–mellow” taste, and a chemically complex aroma matrix [8,9,10,11,12]. Extensive compositional surveys have identified over 1232 volatile organic compounds (VOCs) in Pu-erh tea [11]. This intricate aroma profile is primarily shaped by methoxyphenols, terpenoids, aldehydes, ketones, and esters [10,11,13,14,15], with terpenoids contributing critical floral, woody, and citrus notes depending on their oxidative state [16].

Industrial pile fermentation is the core processing stage governing the formation of these key aroma compounds. This large-scale solid-state process occurs under conditions of high temperature and humidity, fostering an intensive microbial succession and extracellular enzyme secretion within a heterogeneous matrix [1,7,17]. These complex conditions drive systemic biochemical transitions, including polyphenol oxidation, protein hydrolysis, and carbohydrate decomposition, that collectively define the quality formation of RPT [1,8,9]. Consequently, this stage is historically termed the “quality flavor process” [12].

Although the final aroma composition of RPT has been well characterized, a significant gap remains in understanding the kinetic evolution of odor-active compounds during the various stages of industrial pile fermentation. Most existing research has focused on comparing raw materials with final products, lacking a detailed, stage-resolved (e.g., raw material, intermediate, and final fermentation stages) mapping of the chemical transitions that underpin aroma development [11,13,15,18,19,20]. This gap limits a mechanistic understanding of the fermentation process and hinders the development of targeted strategies for aroma quality control and process standardization in industrial production.

To address this, the present study was designed to systematically investigate the stage-dependent evolution of volatile and active aroma compounds during the industrial pile fermentation of RPT. By monitoring two independent production batches and employing headspace solid-phase microextraction coupled with gas chromatography–mass spectrometry (HS-SPME/GC-MS) combined with multivariate statistical analysis and relative odor activity value (rOAV) evaluation, our research specifically aims to provide a comprehensive analysis of the volatile compounds at each stage, providing new insights into the chemical shifts that underpin RPT’s characteristic aroma. The findings are expected to provide a chemical foundation for optimizing fermentation control and enhancing the aroma quality consistency of RPT.

## 2. Materials and Methods

### 2.1. Industrial Fermentation and Tea Sample Collection

Industrially fermented RPT was produced in two independent batches (F1 and F2) at different time points between July and September 2020 by Yunnan Xiaguan Tuocha Tea (Group) Co., Ltd. (100°13′38.755″ E, 25°35′47.342″ N), following an identical traditional spontaneous pile fermentation protocol. A total of 15 tons of sun-dried green tea leaves with an initial moisture content of 8.3% were used as raw materials. To stimulate the proliferation of indigenous microorganisms, the tea leaves were moistened with spring water to reach a moisture content of approximately 40%, and then piled into fermentation heaps with a height of approximately 1.0 m. The piles were turned (broken down, re-moistened, homogenized, and re-piled) every 7–10 days, and a total of eight re-piling operations were performed during the fermentation process. Samples were collected at three key fermentation stages: raw material (RM), intermediate fermentation (IF), and final fermentation (FF). The IF stage was defined as the midpoint of the industrial fermentation process (after the fourth re-piling cycle). A five-point sampling method was employed to minimize spatial heterogeneity within the pile. A total of 18 samples were collected (two batches × three stages × three biological replicates) for VOC analysis (Table S1). Each group of samples was analyzed in triplicate.

### 2.2. Determination of Volatile Compounds in Tea Leaves

#### 2.2.1. Extraction of VOCs

Tea samples were pulverized into a fine powder using liquid nitrogen. A 500 mg aliquot of this powder was transferred into a 20 mL headspace vial (Agilent Technologies, Santa Clara, CA, USA), followed by the addition of a saturated NaCl solution. To improve analytical precision and correct instrumental variability, 10 µL of 3-hexanone-2,2,4,4-d4 (50 µg/mL) was added as an internal standard for semi-quantitative analysis.

#### 2.2.2. HS-SPME Conditions

The headspace vials were equilibrated at 60 °C for 5 min. Subsequently, a 120 µm DVB/CAR/PDMS extraction fiber (Agilent Technologies, Santa Clara, CA, USA) was exposed to the sample headspace for 15 min at 100 °C. To prevent cross-contamination and ensure fiber purity, the fiber was pre-conditioned at 250 °C for 5 min in a conditioning station prior to each analysis.

The HS-SPME procedure was established with reference to commonly applied methods for tea volatile analysis using HS-SPME/GC–MS, with optimized extraction parameters as described above [21].

#### 2.2.3. GC-MS Conditions

Following extraction, the volatile compounds adsorbed on the fiber coating were desorbed in the injection port of an Agilent 8890 GC system (Agilent Technologies, Santa Clara, CA, USA) at 250 °C for 5 min in splitless mode. Identification and semi-quantification were performed using the Agilent 8890 GC coupled with a 7000D MS detector (Agilent Technologies, Santa Clara, CA, USA). Chromatographic separation was achieved on a DB-5MS capillary column (5% phenyl-polymethylsiloxane, 30 m × 0.25 mm × 0.25 µm). Helium served as the carrier gas at a constant flow rate of 1.2 mL/min, with the injector temperature maintained at 250 °C. The oven temperature program was initiated at 40 °C (held for 3.5 min), increased to 100 °C at a rate of 10 °C/min, further elevated to 180 °C at 7 °C/min, and finally reached 280 °C at 25 °C/min, where it was held for 5 min.

Mass spectrometry was operated in electron impact (EI) ionization mode at 70 electron volts. The temperatures of the quadrupole, ion source, and transfer line were maintained at 150 °C, 230 °C, and 280 °C, respectively. Data acquisition was performed in selected ion monitoring (SIM) mode. VOCs were identified by comparison of mass spectra with the NIST library and confirmed using characteristic ions under SIM mode, according to the previously reported method [22].

### 2.3. rOAV Calculation Methods

The rOAVs for each VOC were determined following previously established protocols [23,24]. The compound contributing most significantly to the aroma of the tea sample was assigned an rOAV of 100, and the rOAVs of the remaining compounds were calculated using Equation (1). Generally, VOCs with rOAVs ≥ 1 were considered key aroma contributors, whereas those with 0.1 ≤ rOAVs < 1 were regarded as compounds that contribute to the overall aroma profile of the sample.

(1) $${rOAV}_{i}=\frac{C_{i}}{C_{max}}\times \frac{T_{max}}{T_{i}}\times 100$$

Here, rOAV_i_ signifies the relative odor activity value of any given volatile compound i. *C_i_* and *T*_i_ are the relative content (%) and odor threshold (µg/kg) of compound i, whereas *C_max_* and *T_max_* represent the corresponding relative content and threshold of the compound with the highest aroma contribution.

### 2.4. Statistical Analysis

An HS-SPME/GC-MS analysis of each sample was performed in triplicate. The results are presented as mean values with standard deviations. One-way analysis of variance (ANOVA) followed by Duncan’s multiple range test was employed to assess the statistical significance of differences among samples, with *p* < 0.05 considered statistically significant. Heatmap visualization was constructed using TBtools software version 2.136 (South China Agricultural University, Guangzhou, China). Multivariate statistical analyses were conducted via the Metware Cloud platform (Metware Biotechnology Co., Ltd., Wuhan, China, version 3.0) (https://cloud.metware.cn).

## 3. Results

### 3.1. Overview of Volatile Profiles in RPT During Industrial Pile Fermentation

**Figure 1:**
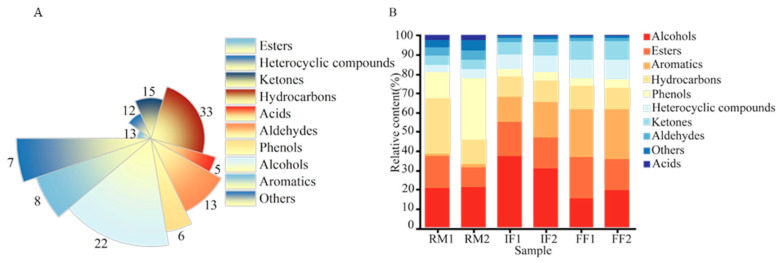
Categories of VOCs in RPT. (**A**) Relative contents of VOCs. (**B**) Relative percentage composition of volatile compound categories across different fermentation stages.

HS-SPME/GC-MS was employed to analyze the VOCs across different fermentation stages of two industrial-scale RPT batches. A total of 134 VOCs were identified (Figure 1A), spanning 9 categories. These included 33 hydrocarbons (24.63%), 22 alcohols (16.42%), 15 ketones (11.19%), 13 esters (9.70%), 13 aldehydes (9.70%), 12 heterocyclic compounds (8.96%), 8 aromatics (5.97%), 6 phenols (4.48%), 5 acids (3.73%), and 7 others (5.22%). Among the 134 VOCs identified in this study, hydrocarbons accounted for the highest proportion (24.63%), followed by alcohols (16.42%) and ketones (11.19%). These findings are in agreement with previous reports, which consistently indicate that hydrocarbons and alcohols are the most predominant classes of VOCs in RPT [11,25].

RM1 was dominated by alcohols and hydrocarbons with moderate ester and phenol relative content. In contrast, RM2 was characterized by the highest relative content of phenols and alcohols, followed by aromatics and hydrocarbons (Figure 1B). During the IF stage, alcohols and esters remained dominant in both batches, while aromatic compounds showed a significant increase, reflecting active biochemical transformations. By the FF stage, esters and aromatics maintained high relative contents, underscoring their pivotal role in shaping the characteristic RPT aroma. Together, the relative content of alcohols declined, while other compounds, including phenols and heterocyclic compounds, exhibited dynamic variations despite their relatively low overall relative content. The observed decrease in alcohols aligns with the findings of Zhao et al. [26], who reported that linalool undergoes biotransformation into terpenols and various oxheterocyclic compounds during the fermentation process. Notably, despite initial differences in raw materials, both batches exhibited analogous aromatic transition patterns during pile fermentation, highlighting the consistency and stability of the industrial process.

### 3.2. Multivariate Statistical Analysis of Stage-Dependent Aroma Evolution

To systematically elucidate the stage-dependent evolution of VOCs during the industrial pile fermentation of RPT, integrated multivariate statistical analyses were performed. Principal Component Analysis (PCA) was first applied to visualize the global trajectory of volatile aroma profile variation. In the diagram (Figure S1), RM1 and RM2 are initially clearly separated. This indicates that differences in fermentation raw materials or time lead to the separation of RM1 and RM2. It is noteworthy that as fermentation proceeds, IF1 and IF2, as well as FF1 and FF2, cluster together respectively. But during the fermentation process, the volatile aroma profiles of the two batches show a similar changing trend. This may be due to the same fermentation process used in the factory, which results in the volatile aroma compounds of the two batches changing in a similar way during fermentation. This convergence indicates that the industrial fermentation process exerts a normalizing effect, driving initially distinct raw materials toward a unified aroma profile.

**Figure 2:**
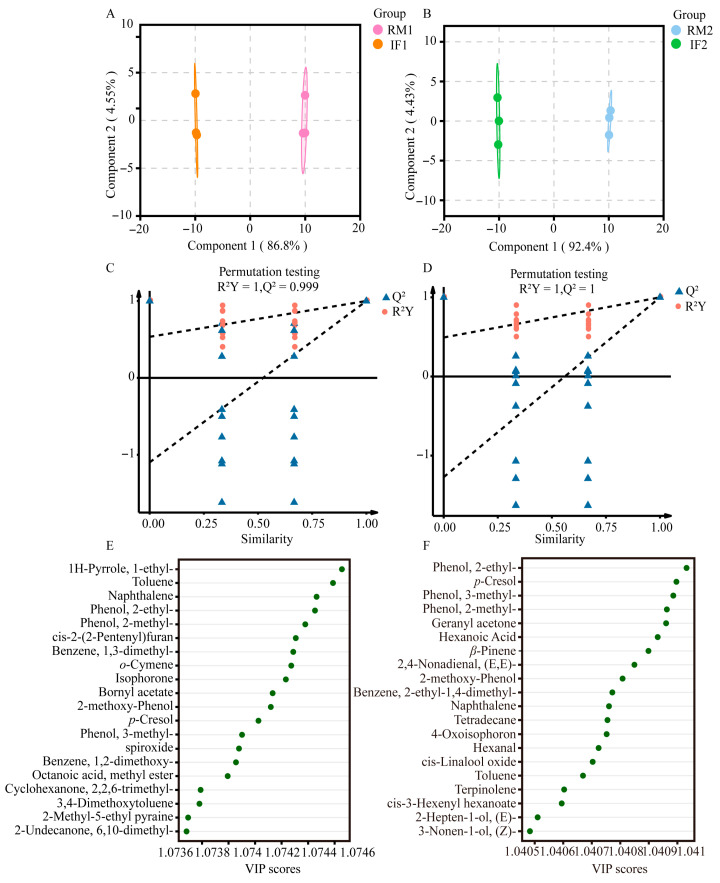
OPLS-DA analysis results of RM vs. IF based on HS-SPME/GC-MS data. (**A**) An OPLS-DA score plot for the comparison of RM1 vs. IF1. (**B**) An OPLS-DA score plot for the comparison of RM2 vs. IF2. (**C**) A cross-validation plot by a 200-times permutation test for the OPLS-DA model comparing RM1 vs. IF1. (**D**) A cross-validation plot by a 200-times permutation test for the OPLS-DA model comparing RM2 vs. IF2. (**E**) Volatiles with VIP > 1.0 identified from the OPLS-DA model comparing RM1 vs. IF1. (**F**) Volatiles with VIP > 1.0 identified from the OPLS-DA model comparing RM2 vs. IF2.

**Figure 3:**
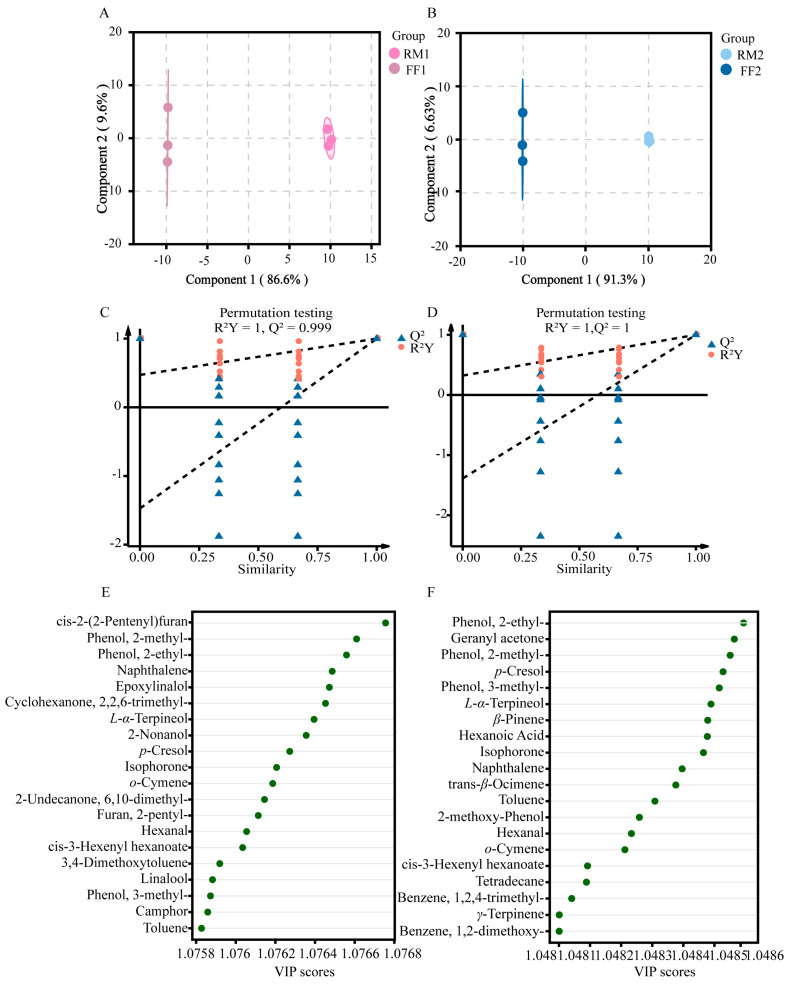
OPLS-DA analysis results of RM vs. FF based on HS-SPME/GC-MS data. (**A**) An OPLS-DA score plot for the comparison of RM1 vs. FF1. (**B**) An OPLS-DA score plot for the comparison of RM2 vs. FF2. (**C**) A cross-validation plot by a 200-times permutation test for the OPLS-DA model comparing RM1 vs. FF1. (**D**) A cross-validation plot by a 200-times permutation test for the OPLS-DA model comparing RM2 vs. FF2. (**E**) Volatiles with VIP > 1.0 identified from the OPLS-DA model comparing RM1 vs. FF1. (**F**) Volatiles with VIP > 1.0 identified from the OPLS-DA model comparing RM2 vs. FF2.

To further screen the compounds driving key stage transitions, four supervised Orthogonal Partial Least Squares Discriminant Analysis (OPLS-DA) models were constructed: (1) IF1 vs. RM1, (2) IF2 vs. RM2, (3) FF1 vs. RM1, and (4) FF2 vs. RM2. Variable Importance in Projection (VIP) values (VIP > 1.0, *p* < 0.05) were used as the primary criterion for identifying significant differential VOCs. In the RM to IF transition, 78 and 85 differential VOCs were identified in Batches 1 and 2, respectively (Figure 2E,F). The top 20 VOCs for IF1 vs. RM1 were primarily nitrogen-containing heterocycles and aromatic hydrocarbons (e.g., 1h-pyrrole,1-ethyl, toluene, naphthalene), along with oxygenated and phenolic compounds (e.g., phenol,2-ethyl-, 2-methyl-phenol, bornyl acetate). For IF2 vs. RM2, phenolics (e.g., phenol,2-ethyl-, *p*-cresol) remained dominant, while fermentation-related terpenes, aldehydes, ketones, and esters (e.g., *β*-pinene, hexanal, hexanoic acid) were also highlighted. Overall, phenolic compounds, aromatic hydrocarbons, and fermentation-derived nitrogen- and oxygen-containing VOCs were key drivers of differentiation. In the FF vs. RM comparisons, 86 and 89 significant differential volatiles were identified for FF1 vs. RM1 and FF2 vs. RM2, respectively (Figure 3E,F). The top 20 for FF1 vs. RM1 included phenolics, oxygenated volatiles, and aromatic hydrocarbons (e.g., phenol,2-methyl-, naphthalene, cis-2-(2-pentenyl)furan, linalool). For FF2 vs. RM2, phenolics (e.g., phenol,2-methyl-, *p*-cresol) were dominant, alongside fermentation-related terpenes, aldehydes, and aromatic hydrocarbons (e.g., *β*-pinene, hexanoic acid, benzene,1,2,4-trimethyl-). Overall, phenolic compounds, terpenes, oxygenated VOCs, and aromatic hydrocarbons were key contributors to volatilome differentiation between fermentation stages.

**Figure 4:**
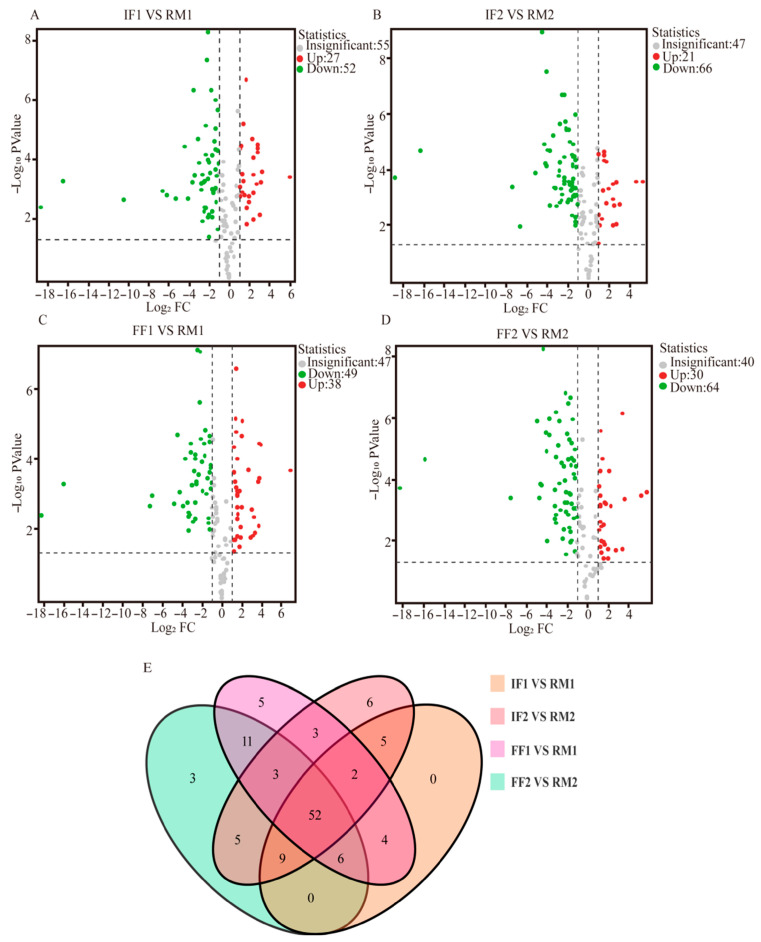
A volcano plot showing the differential volatiles between (**A**) IF1 and RM1, (**B**) IF2 and RM2, (**C**) FF1 and RM1, (**D**) FF2 and RM2; (**E**) Venn diagram of VOCs. (The numbers in each region of the Venn diagram represent the number of compounds identified in the corresponding samples. Numbers in overlapping regions indicate compounds shared by the intersecting groups, whereas numbers in non-overlapping regions represent compounds unique to a specific group).

To quantitatively characterize VOC accumulation directions and class-level tendencies, a volcano plot analysis was conducted for intermediate and final transitions (Figure 4A–D). The volcano plot analysis revealed significant alterations in the metabolite profiles of RPT after industrial pile fermentation. In Batch 1, 27 metabolites were significantly increased in IF1 compared with RM1, mainly esters (5), aldehydes (7), and hydrocarbons (7), whereas 52 metabolites were decreased, predominantly hydrocarbons (15), aldehydes (7), ketones (5), heterocyclic compounds (5), and phenols (6). In Batch 2, 21 metabolites increased, including esters (5), alcohols (5), and aromatics (3), while 66 metabolites decreased, chiefly hydrocarbons (17), ketones (7), heterocyclic compounds (7), aldehydes (7), and phenols (6), indicating an overall reduction in metabolite content after fermentation, with hydrocarbons being the most affected class in both batches. Notably, alcohols displayed batch-dependent behavior, decreasing in IF1 but increasing in IF2, reflecting the complex and variable influence of fermentation. In the FF samples, however, alcohols showed a consistent increase in both batches (11 in FF1 and 9 in FF2), along with significant changes in hydrocarbons, ketones, and heterocyclic compounds, highlighting reproducible metabolic shifts induced by the fermentation process.

To further identify VOCs consistently altered across both batches and stage transitions, a Venn diagram intersection analysis was performed (Figure 4E). Through the Venn diagram analysis, it can be intuitively observed that 52 compounds were common differential volatile compounds in the first and second batches of Pu-erh tea at different fermentation stages, mainly hydrocarbon compounds, indicating that hydrocarbon compounds play an important role in the aroma of ripe tea during fermentation, which was highly consistent with the conclusion of “thermal immobilization-dominated metabolic recombination” in the study of mandarin tea [27]. Both studies have demonstrated that high-temperature and high-humidity conditions serve as the primary drivers of aroma precursor transformation. In addition, there were 5 unique differential volatile compounds in IF1 vs. RM1 and IF2 vs. RM2 that collectively contributed to fresh aroma attenuation, primarily including pyrazine, 2,5-dimethyl- (roasted); 1,5-heptadien-4-one, 3,3,6-trimethyl- (herbal, honey, minty, berry); *β*-guaiene (sweet, woody); 1-hexanol, 2-ethyl- (fruity); and benzene, 1,2,4,5-tetramethyl- (sweet, rancid). By contrast, 11 unique differential volatile compounds in FF1 vs. RM1 and FF2 vs. RM2 imparted dominant woody and caramel–sweet characteristics, defining the final aroma profile. These primarily included 2h-pyran-3-ol, 6-ethenyltetrahydro-2,2,6-trimethyl- (floral, honey); pyrazine, methyl- (nutty, roasted, chocolate, green); 1-(2-Hydroxyethyl)-1,2,4-triazole; trans-*β*-ocimene (sweet, herbal); *β*-myrcene (floral, fruity); *β*-ocimene (fruity); benzeneacetaldehyde (floral, honey, cherry); *L*-*α*-terpineol (pine); linalool (floral, green); 1-nonanol (floral); and 2-Nonanol (rose).

### 3.3. Identification of Key Aroma-Active Compounds Based on rOAVs

**Figure 5:**
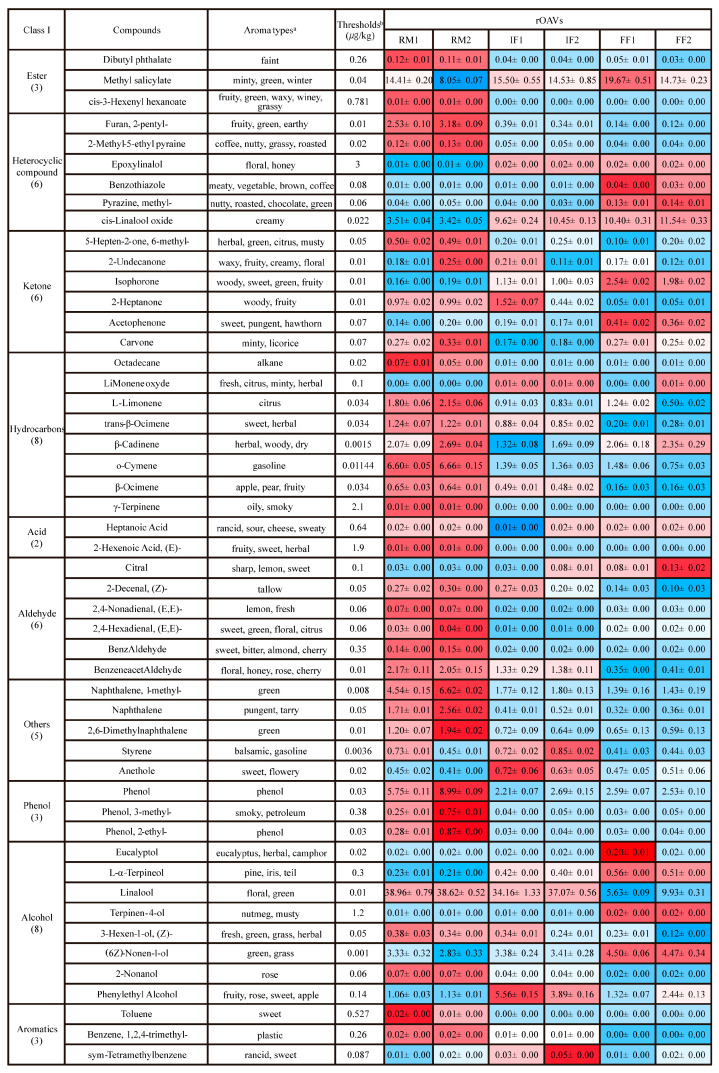
Aroma types and rOAVs of VOCs. ^a^ Aroma types were categorized based on references [11,32] and a database (https://www.thegoodscentscompany.com/search.php/, accessed on 20 October 2025). ^b^ Odor thresholds were obtained from reference [32] and a database (https://www.vcf-online.nl/, accessed on 22 October 2025). Heatmap colors represent rOAVs values: red indicates higher values, blue indicates lower values. Values are shown as mean ± SD.

The characteristic aroma of a sample is determined by both the content and odor threshold of individual VOCs. The odor threshold refers to the minimum concentration at which a volatile organic compound can be detected by human perception. rOAVs were calculated to evaluate the impact of individual VOCs on the overall tea aroma, where rOAVs > 1 indicates a significant contribution [28]. Based on the selection criteria of VIP > 1 and *p* < 0.05, the rOAVs of 50 VOCs were determined and visualized (Figure 5). Among these, 13 VOCs with rOAVs > 1 were identified as primary contributors. These compounds span eight chemical classes: esters (1), heterocyclic compounds (2), ketones (1), hydrocarbons (3), aldehydes (1), phenols (1), alcohols (3), and others (1). Consequently, these 13 VOCs were determined to be the core constituents responsible for the enhanced aroma profile of fermented RPT.

### 3.4. Dynamic Transition of Aroma Characteristics: From Fresh–Green to Woody–Minty

The dynamic changes in rOAVs revealed stage-dependent changes during Pu-erh tea pile fermentation. During the early fermentation stage (from RM to IF), the compounds associated with fresh aromas exhibited an overall downward trend: among them, rOAVs of linalool (floral aroma) decreased from 38.96 to 34.16 in Batch 1 and from 38.62 to 37.07 in Batch 2; those of *D*-limonene (with a citrusy aroma) dropped from 1.80 to 0.91 in Batch 1 and from 2.15 to 0.83 in Batch 2; and those of furan, 2-pentyl (green aroma) declined from 2.53 to 0.39 in Batch 1 and from 3.18 to 0.34 in Batch 2. This attenuation weakened the tea’s initial floral, citrus, and green aroma characteristics.

During the late fermentation stage (from IF to FF), the rOAVs of isophorone (woody aroma) increased from 1.13 to 2.54 in Batch 1 and from 1.00 to 1.98 in Batch 2. In contrast, methyl salicylate (with a minty aroma) rose from 15.50 to 19.67 in Batch 1, whereas only a negligible increase was observed in Batch 2. Concurrently, residual linalool (floral aroma) further decreased to 5.63 in Batch 1 and 9.93 in Batch 2. The decrease in floral substances such as linalool during the RM to IF stage may relate to the thermal degradation of floral precursors [27]. It was confirmed that the thermal treatment of mandarin tea reduced flavonoid glycosides, potential precursors of floral volatiles. Isophorone accumulation in the IF to FF stage is associated with woody aroma, and isophorone was identified as a core contributor to woody aroma in yeast-enhanced fermentation, with its content positively correlating to sensory “woody aroma intensity” [21]. This dynamic shift transitioned the aroma profile from fresh to dominant woody and minty characteristics. It is noteworthy that methoxyphenolic compounds, which are widely recognized as major contributors to the stale aroma of RPT [18,29,30], were not detected in this study. This absence may be attributed to variations in detection methods or differences in the composition of active microbial communities during fermentation. Methoxyphenols are typically linked to the metabolic activities of specific microbial strains [31] and therefore, discrepancies in microbial activity across different production batches could explain their absence. Further research is needed to explore how microbial diversity influences the biosynthesis of these aroma-active compounds.

### 3.5. Construction of an Industrial RPT Flavor Wheel

**Figure 6:**
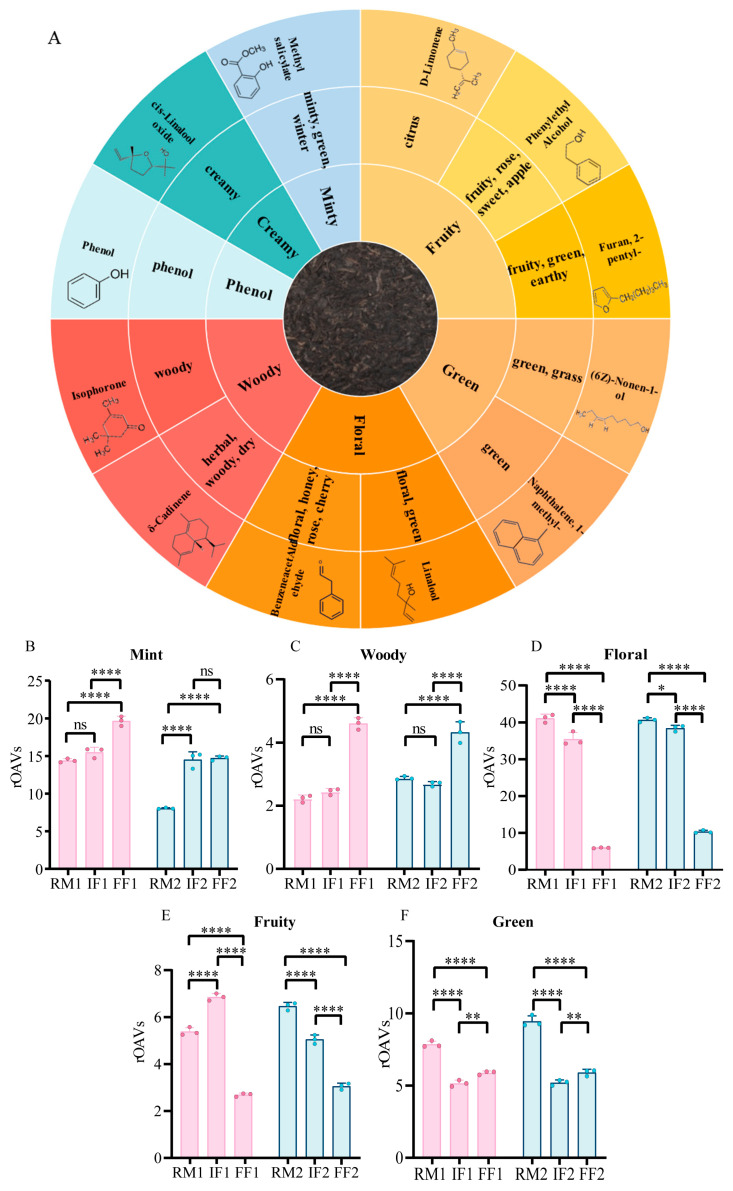
(**A**) Flavor wheel representing the characteristic VOCs in RPT. Different colors represent different aroma categories. Each segment shows specific aroma descriptors corresponding to the category. (**B**–**F**) Cumulative rOAVs of VOCs categorized by distinct aroma types. Red represents the cumulative rOAVs of VOCs across different aroma categories in the first batch of tea samples, while blue represents those in the second batch.(* *p* < 0.05, ** *p* < 0.01, **** *p* < 0.0001, ns: not significant).

To characterize the unique aromatic profile of industrial RPT, a flavor wheel was developed based on the aroma categories of VOCs with rOAVs > 1 (Figure 6A). These key volatile components were prioritized using the criteria of VIP > 1 and *p* < 0.05 to ensure their significance to the overall aroma. The resulting flavor wheel illustrates that the characteristic aroma of RPT is primarily composed of 7 distinct sensory attributes: minty, woody, floral, fruity, green, phenol, and creamy.

The first group of compounds was characterized by a woody aroma, including isophorone (average rOAVs = 1.17) and *δ*-cadinene (average rOAVs = 2.03). Isophorone was determined to be the major volatile compound present in pu-erh tea [11,32]. The second group comprised compounds with a minty aroma, represented by methyl salicylate (average rOAVs = 14.48). Methyl salicylate is a characteristic volatile compound synthesized from salicylic acid under the catalysis of salicylic acid hydroxyl methyltransferase; it is widely distributed in various tea species and serves as a key contributor to the minty aroma [33]. Methyl salicylate has been reported to originate from the enzymatic hydrolysis of its corresponding primeveroside precursor [34]. The third group exhibited a fruity aroma, including *D*-limonene (average rOAVs = 1.24), phenylethyl alcohol (average rOAVs = 2.57), and furan, 2-pentyl- (average rOAVs = 1.12). The fourth group contained compounds with a floral aroma, such as benzeneacetaldehyde (average rOAVs = 1.28) and linalool (average rOAVs = 27.39). Linalool is an important volatile constituent in RPT, generated from geranyl pyrophosphate precursors through the catalytic action of linalool synthase [35]. Additionally, we identified *(6Z)*-nonen-1-ol (green aroma, average rOAVs = 3.65), naphthalene, 1-methyl- (green aroma, average rOAVs = 2.93), phenol (phenol aroma, average rOAVs = 4.13), and cis-linalool oxide (creamy aroma, average rOAVs = 8.16).

The comparison of rOAVs of characteristic VOCs with different aroma types in each batch showed that the cumulative rOAVs of the RM group, IF group and FF group were different (Figure 6B–F). Interestingly, the change trend of the minty, woody, floral, fruity and green aroma of the two batches of industrial fermented tea samples is roughly the same. The content of minty aroma and woody aroma increased significantly after pile fermentation, and the content of flower aroma and green and fruity aroma decreased significantly after pile fermentation.

## 4. Discussion

The present study focused on elucidating the dynamic evolution of VOCs during the industrial pile fermentation of RPT using two independent production batches from the same manufacturer. The reproducible stage-dependent changes observed across both batches highlight the robustness of standardized industrial fermentation in shaping aroma characteristics. Despite minor initial differences in raw materials, the volatile profiles converged toward the final fermentation stage, suggesting that controlled pile fermentation exerts a homogenizing effect on aroma composition. This phenomenon indicates that process parameters such as temperature regulation, moisture control, and turning frequency may play a dominant role in determining the final sensory profile under industrial conditions.

It should be acknowledged that RPT produced by different manufacturers or under traditional fermentation systems may exhibit compositional variations. Factors such as raw material origin, microbial community structure, fermentation duration, and environmental conditions can influence volatile formation pathways [29,31,36,37]. For example, certain methoxyphenolic compounds often associated with aged RPT aroma were not prominent in the present samples, which may reflect differences in microbial ecology or processing parameters specific to the industrial system examined. Comparative investigations involving multiple production sites or traditional fermentation models would further clarify the extent to which the observed aroma evolution patterns represent universal characteristics of RPT versus process-specific outcomes.

In addition to sensory attributes, RPT is widely recognized for its functional and health-related properties [38]. Although the present study primarily addressed aroma-active VOCs, fermentation is also known to modulate non-volatile bioactive constituents [39]. In our previous investigation conducted on the same industrial fermentation batches, comprehensive lipidomic analysis revealed the substantial remodeling of lipid subclasses and medium- to long-chain fatty acids during fermentation, accompanied by dynamic shifts in polyphenol-related compounds [40]. These transformations suggest that industrial pile fermentation does not exclusively drive volatile formation but instead induces broader metabolic reorganization within the tea matrix. The coexistence of volatile evolution and lipid remodeling implies potential biochemical linkages, as lipid degradation and oxidative processes may contribute to the generation of certain aroma precursors. Therefore, the quality formation of RPT should be considered as a coordinated transformation of both sensory and functional components rather than isolated chemical events.

Overall, the findings of this study provide a process-oriented framework for understanding aroma development under standardized industrial conditions while acknowledging potential variability arising from different production contexts. Together with previous compositional evidence from the same fermentation system, the present results support the concept that industrial pile fermentation orchestrates systematic chemical transitions that underlie both aroma formation and functional component transformation. Such insights contribute to improving aroma quality control and promoting further integrative research in RPT fermentation science.

## 5. Conclusions

This study provides a detailed characterization of the stage-dependent evolution of volatile and active aroma compounds in RPT during industrial pile fermentation. Through the use of HS-SPME/GC-MS, multivariate statistical analysis, and rOAV evaluation, we identified 134 VOCs, with hydrocarbons, alcohols, and esters as the predominant classes. In total, 13 key aroma-active compounds (rOAVs > 1) were found to drive RPT’s unique aroma profile. A significant shift in aroma profiles was observed: fresh and green aromas (e.g., linalool, *D*-limonene) decreased in early fermentation, while woody and minty aromas (e.g., isophorone, methyl salicylate) became more pronounced in the later stages. These findings provide mechanistic insights into the transformation of aroma from fresh to woody and minty, a critical aspect of aroma formation not fully explored in previous research. Additionally, the flavor wheel developed from the rOAV results visually demonstrates the dynamic aroma transitions during fermentation. This study enhances our understanding of aroma dynamics in RPT and offers valuable insights for improving aroma quality control and standardizing RPT production processes.

## Data Availability

The original contributions presented in this study are included in the article/Supplementary Materials. Further inquiries can be directed to the corresponding author.

## Supplementary Materials

The following supporting information can be downloaded at https://www.mdpi.com/article/10.3390/foods15061014/s1, Figure S1: PCA results obtained via HS-SPME/GC-MS; Table S1: Tea leaf samples collected during pile fermentation of Pu-erh tea.
