# Supplementary material for: Dynamic Evolution of Aroma Characteristics in Ripened Pu-Erh Tea During Industrial Fermentation: Insights from GC-MS and Flavor Wheel Analysis
Source: Foods. 2026 Mar 13;15(6):1014. doi: 10.3390/foods15061014 (PMC13025987; doi:10.3390/foods15061014)
Supplement: Supplementary file 1 [file foods-15-01014-s001.zip › foods-4163672-supplementary.pdf]

Table S1. Tea leaf samples collected during pile-fermentation of Pu-erh tea.

	Fermentation 1		Fermentation 2	
	Collection Date (2020)	Sample ID	Collection Date (2020)	Sample ID
Raw martial(RM)	01/07	RM1-1, RM1-2, RM1-3	15/07	RM2-1, RM2-2, RM2-3
Intermediate fermenting sample(IF)	29/07	IF1-1, IF1-2, IF1-3	13/08	IF2-1, IF2-2, IF2-3
Final fermented sample(FF)	24/08	FF1-1, FF1-2, FF1-3	08/09	FF2-1, FF2-2, FF2-3

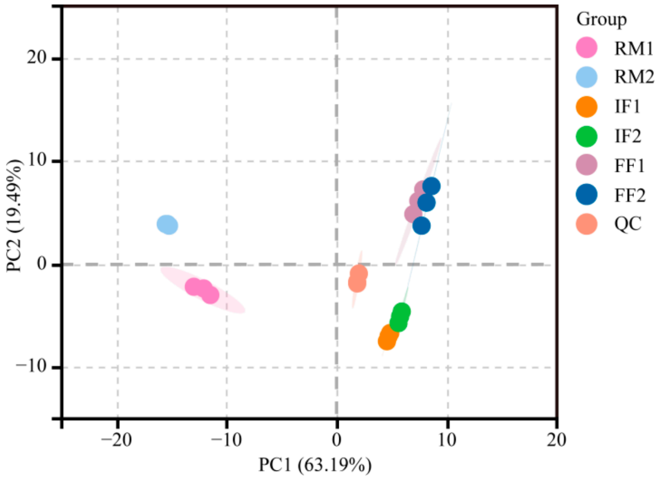

Figure S1. PCA results obtained via HS-SPME/GC-MS.
